# Mapping analysis to predict EQ-5D-5 L utility values based on the Oxford Hip Score (OHS) and Oxford Knee Score (OKS) questionnaires in the Spanish population suffering from lower limb osteoarthritis

**DOI:** 10.1186/s12955-020-01435-8

**Published:** 2020-06-15

**Authors:** Jesús Martín-Fernández, Mariel Morey-Montalvo, Nuria Tomás-García, Elena Martín-Ramos, Juan Carlos Muñoz-García, Elena Polentinos-Castro, Gemma Rodríguez-Martínez, Juan Carlos Arenaza, Lidia García-Pérez, Laura Magdalena-Armas, Amaia Bilbao

**Affiliations:** 1 Unidad Docente Multiprofesional de Atención Familiar y Comunitaria Oeste, Gerencia Asistencial de Atención Primaria, Servicio Madrileño de Salud, Móstoles, Madrid Spain; 2 Facultad de Ciencias de la Salud, Universidad Rey Juan Carlos, Alcorcón, Madrid Spain; 3 Red de Investigación en Servicios Sanitarios y Enfermedades Crónicas (REDISSEC), Madrid, Spain; 4 Unidad de Apoyo a la Investigación, Gerencia Asistencial Atención Primaria, Madrid, Spain; 5 Dirección General de Salud Pública, Consejería de Sanidad Comunidad de Madrid, Madrid, Spain; 6 Programa de doctorado Epidemiologia y Salud Publica, Universidad Rey Juan Carlos, Alcorcón, Madrid Spain; 7 C.S. San Martín de Valdeiglesias, Gerencia Asistencial de Atención Primaria, Servicio Madrileño de Salud, San Martín de Valdeiglesias, Madrid Spain; 8 C.S. Alcalde Bartolomé González, Gerencia Asistencial de Atención Primaria, Servicio Madrileño de Salud, Móstoles, Madrid Spain; 9 C.S. El Soto. Gerencia Asistencial de Atención Primaria, Servicio Madrileño de Salud, Móstoles, Madrid Spain; 10 Unidad Docente Multiprofesional de Atención Familiar y Comunitaria Norte, Gerencia Asistencial de Atención Primaria, Servicio Madrileño de Salud, Madrid, Spain; 11 C.S. Infante Don Luis, Gerencia Asistencial de Atención Primaria, Servicio Madrileño de Salud, Boadilla del Monte, Madrid Spain; 12 Osakidetza, Hospital Universitario Basurto, Servicio de Traumatología y Cirugía Ortopédica, Bilbao, Bizkaia Spain; 13 Red de Investigación en Servicios Sanitarios y Enfermedades Crónicas (REDISSEC), Bilbao, Bizkaia Spain; 14 Fundación Canaria Instituto de Investigación Sanitaria de Canarias (FIISC), La Laguna, Santa Cruz de Tenerife Spain; 15 Red de Investigación en Servicios Sanitarios y Enfermedades Crónicas (REDISSEC), El Rosario, Santa Cruz de Tenerife Spain; 16 Hospital Universitario Nuestra Señora de Candelaria, El Rosario, Santa Cruz de Tenerife Spain; 17 Unidad de Investigación, Hospital Universitario Basurto, Osakidetza, Bilbao, Bizkaia Spain; 18 Instituto de Investigación en Servicios de Salud Kronikgune, Barakaldo, Bizkaia Spain

**Keywords:** Quality of life, Health status, Osteoarthritis, Economics, Cost-benefit analysis, EQ-5D

## Abstract

**Background:**

The EQ-5D-5 L is a quality-of-life questionnaire based on individuals’ preferences that is widely employed for cost-effectiveness analysis. Given the current demand for mapping algorithms to directly assign “utilities”, this study aimed to generate different mapping models for predicting EQ-5D-5 L utility values based on scores of the Oxford Hip Score (OHS) and Oxford Knee Score (OKS) questionnaires provided by patients suffering from hip and knee osteoarthritis (OA), respectively, and to assess the predictive capability of these functions.

**Methods:**

This was a prospective, observational study. Following the criteria of the American Rheumatism Association, 361 patients with hip OA and 397 with knee OA from three regions in Spain were included. Health-related quality of life (HRQoL) was assessed through the EQ-5D-5 L general questionnaire and the OHS and OKS specifically for lower limb OA.

Based on the scores on the OHS and OKS questionnaires, EQ-5D-5 L utilities were estimated using 4 models: ordinary least squares (OLS), Tobit, generalized linear model (GLM), and beta regression (Breg).

The models were validated on the same patients after 6 months: the mean absolute error (MAE) and mean squared error (MSE) with their 95% confidence intervals (CI), mean values of standard errors (SE), intraclass correlation coefficients (ICC), and Bland-Altman plots were obtained.

**Results:**

The lowest MAEs were obtained using GLM and Breg models, with values of 0.1103 (0.0993–0.1214) and 0.1229 (0.1102–0.1335) for hip OA, and values of 0.1127 (0.1014–0.1239) and 0.1141 (0.1031–0.1251) for knee OA. MSE values were also lower using GLM and Breg. ICCs between predicted and observed values were around or over the 0.8 cut-off point. Bland-Altman plots showed an acceptable correlation, but precision was lower for subjects with worse HRQoL, which was also evident when comparing MAEs of the bottom and top halves of the utilities scale. Predictive equations for utilities based on OHS/OKS scores were proposed.

**Conclusions:**

The OHS and OKS scores allow for estimating EQ-5D-5 L utility indexes for patients with hip and knee OA, respectively, with adequate validity and precision. GLM and Breg produce the best predictions. The predictive power of proposed equations is more consistent for subjects in better health condition.

## Background

Knowing citizens’ and patients’ perceptions of health-related quality of life (HRQoL) is essential for assessing health interventions and formulating healthcare policies. Tools that measure HRQoL based on patient preferences are indispensable [1]. These tools allow individuals to express the impact of poor health on their lives and their preferences for certain health states. These preferences can be characterized as “utilities”, a measure of the strength of a person’s preference for a specific health state in relation to alternative health states. The utility scale assigns numerical values on a scale from 0 (death) to 1 (optimal or ‘perfect’ health). Health states can be considered worse than death and thus have a negative value. Health state preference scores can be transformed into quality-adjusted life years (QALYs), which are an outcome metric for health benefit used in many health economic evaluations [2].

Utility values can be obtained via different methods, some of which are direct, such as the Time Trade Off (TTO) or Standard Gamble methods [3]. However, due to the complexity of these tasks, a preference-based measure is often implemented instead. One frequently used preference-based measure is the EQ-5D. The utility values, known as EQ-5D index scores, represent the preferences of the general population over these health states as defined by the EQ-5D, which are collected through a large population survey based on the TTO method [4]. When studies do not have preference measures available, these data can be estimated by “mapping” other HRQoL measures or health-related benefits observed in the relevant clinical trial(s) to the known preference measure, i.e., the EQ-5D [1, 5, 6].

“Mapping” implies developing and employing an algorithm (or algorithms) for the prediction of specific outcomes (named “health utilities”) that express general preferences obtained from other indicators or health measures. The predictive measure of the utilities can be an indicator of a health outcome that is not based on preferences [7]. The EQ-5D is a quality-of-life questionnaire based on individuals’ preferences and is the most widely employed for cost-effectiveness analysis of healthcare technologies [8] and by some European organizations for technology evaluation, such as the National Institute for Health and Care Excellence (NICE) in the United Kingdom [9]. In Spain, the utilities scores derived from the last version of EQ-5D, the ED-5D-5 L, have been proposed to inform Spanish health technology assessments [10]. For these reasons, there is substantial demand for mapping algorithms that employ statistical analysis of answers or scores obtained with tools not susceptible to directly assigning “utilities” for the prediction of EQ-5D utility indexes [5, 6].

Measuring the impact of disease on quality of life and the effect of interventions on disease is especially important in the case of conditions that cause chronic deterioration of health at the population level. Lower limb (hip and knee) osteoarthritis (OA) is a very prevalent disease that places a great burden on the individual and the society, worldwide and specifically in Spain. OA is the 11th cause of impairment in the world, with a prevalence standardized by age of 3.8 and 0.9% for knee and hip OA, respectively. Disability-adjusted life years (DALYs) resulting from it increased 70% over the last 20 years [11]. Lower limb OA has shown a great impact on HRQoL in Spanish population [12], and it has been estimated a yearly cost of €1500 per patient with knee or hip OA in 2007, 86% of which were direct costs [13]. The current cost of generalized OA healthcare is set between 0.25 and 0.50% of Spain’s GDP [14].

There are several questionnaires, adapted and validated for Spain, to assess HRQoL in patients with lower limb OA, such as the Western Ontario and McMaster Universities Osteoarthritis Index (WOMAC) [15, 16]. The Hip Outcome Score (HOS) has shown its usefulness for patients about to undergo hip arthroscopy [17, 18]. Other questionnaires, such as the Knee Society Clinical Rating System (KSS) [19, 20] and the Knee Injury and Osteoarthritis Outcome Score (KOOS) [21, 22], specifically assess HRQoL in patients with knee OA. The Oxford Hip Score (OHS) [23] and Oxford Knee Score (OKS) [24] are questionnaires designed to assess the outcome following hip or knee replacement; they have recently been validated in Spanish for the population in Spain suffering from hip or knee OA whether or not undergoing surgical procedures [25, 26]. Although OHS and OKS scores do not inform about preferences on the health states, mapping procedures have been developed to predict utilities based on EQ-5D using scores from the OKS and OHS in other countries [27, 28].

This study aims to assess different mapping models that employ OHS and OKS scores reported by patients with hip and knee OA, respectively, for predicting utility values assigned by the EQ-5D-5 L questionnaire to particular health conditions, as well as assessing the predictive capability of these utility indexes.

## Methods

### Design

This was an observational study with a 6-month follow-up period. OHS and OKS scores and EQ-5D responses at inclusion made up the estimation sample. The validation sample comprised patient responses after the follow-up period.

### Sampling and sample size

Opportunistic sampling was performed. Patients > 18 years of age diagnosed with hip or knee OA according to the criteria by the American Rheumatism Association [29, 30] were recruited from traumatology, rheumatology, and primary care consultations in Vizcaya, Madrid, and Tenerife, three very different areas in Spain. Participants were added to the study consecutively between January and December 2015. Patients who did not understand Spanish, were not able to read or write, or were diagnosed with a cognitive impairment were excluded. All the patients provided written consent to participate in the study, and the relevant Ethics Committees for Clinical Research granted approval.

The sample size was calculated for ordinary least squares (OLS) models as these models present sufficient demands to provide us with an adequate sample size. An OLS predictive model is considered to have sufficient predictive power when R^2^ ≥ 0.50, for 300 subjects and 15 predictive variables [31]. The OKS and OHS questionnaires comprise 12 items, so if we were to recruit at least 300 subjects, the sample size would be sufficient, even including age and sex as predictive variables.

### Variables

The following data were collected for all the patients: age, gender, body mass index, arthritis-affected joints, previous joint replacement surgeries, and comorbidity as measured via the Charlson index [32]. All the patients completed the EQ-5D-5 L questionnaire [33], which comprises two parts. The first part consists of 5 questions on the individual’s health condition in terms of mobility, self-care, daily life performance, pain/discomfort, and anxiety/depression. Each dimension was measured on a 5-point scale, and a single weighted score (the utility index) was drawn, so that the higher the score was, the better the health status was. Utility values were derived from the algorithm proposed for the Spanish population (ranging from − 0.4162 to 1) [10]. The second part of the EQ-5D-5 L questionnaire consists of a visual analogue scale (VAS), which was not employed in this study.

Patients with hip and knee OA completed the Spanish (Spain) version of the OHS [23, 25] and OKS [24, 26], respectively. Both questionnaires are self-administered questionnaires that can be answered via “face-to-face” interviews or mailed in by the patient after completion. They include 12 questions with 5 possible answers for the assessment of HRQoL as perceived by the patient over the last 4 weeks, covering pain, mobility and ability to carry out regular tasks. Each question is given a score of 0 to 4, with the latter being the best possible outcome. The final score is calculated by summing up the individual scores and ranges from 0 to 48, with 48 the best possible outcome [34]. The scores were developed to assess the outcome of hip and knee replacements, but they have also been used to assess changes in the basal situation of a patient with hip or knee OA [25, 26, 34]. The questionnaires were completed at the clinic, after the inclusion of patients and at the 6-month follow-up visit (Additional file 1 shows all the questions in the Spanish (Spain) version of the OHS, and Additional file 2 shows all the OKS questions in its Spanish (Spain) version).

### Statistical analysis

Explanatory and dependent variables were described by descriptive statistical analysis and correlations between general and disease-specific measurements of HRQoL.

### Statistical models

We estimated direct utility mapping models by regressing responses to individual OKS/OHS questions directly onto EQ-5D utility using four different models.

First, an exploratory Ordinary Least Squares (OLS) model was conducted in the estimation sample. The selected dependent variable was from the EQ-5D-5 L, and total OHS/OKS score was the only regressor to test the degree of correspondence between the two measures [7].

Afterwards, the following 4 regression models were employed to estimate the EQ-5D-5 L utility values based on the items in the OHS and OKS questionnaires:

1. OLS model. This method assumes that EQ-5D-5 L scores can be predicted as a linear combination of the answers to the OHS or OKS.
2. Tobit regression models. This type of model has been proposed as useful for assessing the relationship between health factors and continuous measures of quality of life and, under certain circumstances, is able to circumvent the ceiling effect bias in health measurements [35]. The dependent variable (utility) was censored at values of − 0.4162 and 1, respectively, which makes this method appropriate [36].
3. Generalized linear models (GLM). The chosen dependent variable was disutility (disutility = 1 - utility), which allows for overcoming the biased distribution of utility values and the prediction of disutilities > 1 [27]. The logarithmic function was chosen as the link function and the Gaussian family selected as the distribution family since they provided the most adequate measurements of goodness-of-fit, according to the Akaike Information Criteria (AIC) and Bayes Information Criteria (BIC).
4. Beta regression (Breg) models provide flexible approaches to regress the outcomes with truncated supports, such as HRQoL, on covariates, after accounting for different characteristics of the outcome distribution [37]. Beta regression is a model of the mean of the dependent variable *y* conditional on covariates *x*. Beta regression is only appropriate for a dependent variable that is strictly greater than 0 and strictly less than 1; as a result, we had previously transformed any value for utilities *y* in *y’* where:

$$ y\hbox{'}=\left[y-\left(\mathrm{minimum}\ \mathrm{value}\right)\right]/\left[1-\left(\mathrm{minimum}\ \mathrm{value}\right)\right] $$

To obtain an open (0,1) interval, we transformed boundary points to slightly greater or smaller values by applying the formula ([y’(10^5^–1) + 0.5])/ 10^5^, where y’ is the dependent observed variable in the [0,1]. This measure was supposed to increase/decrease these values by less than 10^− 5^.

The conditional mean, the utility estimation, should also be in (0, 1). This is accomplished by using the logit as the link function for the conditional mean. One main difference from the logistic regression model is that there is no need for responses to be dichotomous (the transformed utility values are continuous). Beta regression was estimated by maximum likelihood methods, and variance was directly estimated from the data.

Predictive models were built using function (1) for OLS, function (2) for Tobit models, function (3) for GLM (which used the logarithm function as the link function), and (4) for beta regression:

1 $$ {\hat{U}}_i={\beta}_0+{\beta}_i{x}_i $$

2 $$ {\displaystyle \begin{array}{l}-0.4162\ \mathrm{if}\ {\hat{U}}_i\le -0.4162\\ {}{\hat{U}}_i={\beta}_0+\beta {\hbox{'}}_i{x}_i\\ {}\mathrm{Where}\ {\beta}_i^{\hbox{'}}={\beta}_i\cdot \times \left[1-\frac{\alpha_l{f}_l-{\alpha}_u{f}_u}{F_l-{F}_u}-{\left(\frac{f_l-{f}_u}{F_l-{F}_u}\right)}^2\right]\\ {}{\upalpha}_{\mathrm{l}}=\left(\mathrm{l}-{\mathrm{x}}_{\mathrm{i}}{\upbeta}_{\mathrm{i}}\right)/\upsigma; \kern0.75em {\upalpha}_{\mathrm{u}}=\left(\mathrm{u}-{\mathrm{x}}_{\mathrm{i}}{\upbeta}_{\mathrm{i}}\right)/\upsigma; \kern0.5em \mathrm{l}=-0.4162;\mathrm{u}=1\\ {}\mathrm{f}\left(\mathrm{z}\right):\mathrm{standard}\ \mathrm{normal}\ \mathrm{density},\\ {}\mathrm{F}\left(\mathrm{z}\right):\mathrm{cumulative}\ \mathrm{normal}\ \mathrm{distribution}\ \mathrm{function}\\ {}1\ \mathrm{if}\;{\hat{U}}_i\ge 1\end{array}} $$

3 $$ {\hat{U}}_i=1-{e}^{\beta 0+\beta ixi} $$

4 $$ {\hat{U}}_i=\frac{{{e^{\beta}}^0}^{+\beta ixi}}{1+{e}^{\beta 0+\beta ixi}}, $$

where ${\hat{U}}_i$ stands for the estimation of utilities, β_0_ is the constant term, β_i_ is the vector of the regressors of each model, and x_i_ is the value of the selected variables from the OHS and OKS in the derivation model.

Using a two-part model, as proposed by many authors [1], was ruled out since only 2.5% of the patients expressed the maximum utility level at the time of inclusion.

To build the models, all the OHS and OKS questions were initially included as independent variables, and coefficients whose significance threshold was less than 0.1 were selected for the final model. OHS/OKS responses are ordinal, but they can be treated as continuous variables under the assumption that they indicate levels of clinical severity [34]. Consequently, models were tested with the questions from the OHS/OKS questionnaires treated as ordinal and as continuous variables. Age and sex were included as predictive variables in the preliminary tests.

### Evaluation of models

To study the adequacy of the models, the distribution of residuals was assessed. Additionally, the coefficients of determination (R^2^ or pseudoR^2^) were studied for the OLS and Tobit models following the BIC and AIC.

Standard error (SE) of the coefficients were calculated using robust methods to prevent the presence of heteroscedasticity since the patients all came from different consultations (clusters) [1, 38].

The intraclass correlation coefficient (ICC, two random factors, absolute agreement) was used to test the relation between predicted and observed values in the estimation and validation samples. The mean absolute error (MAE), which is the mean value of the absolute differences between observed and predicted EQ-5D-5 L utilities, and the mean squared error (MSE), which is the average of the squares of errors, were calculated to assess the predictions of each model for both the estimation and validation samples. Following standard recommendations, the mean of the SE is also presented for each model as a measure of individual variability of the prediction [7]. All these measures were compared for utilities above and below the median to evaluate the fitting of the models in patients with better and worse HRQoL reported.

Additionally, Bland-Altman [39] plots were generated to ascertain the agreement between observed and predicted values in the validation sample.

Stata 14.0® software was used to perform the statistical analysis.

A statement on adherence of the manuscript to MAPs [7] is presented in Additional file 3.

## Results

The study included 361 patients diagnosed with hip OA and 397 with knee OA, of whom 356 and 391 subjects completed the questionnaire at the inclusion visit, respectively. These subjects made up the estimation sample. For the OHS survey, questions 3 and 4 were answered in all cases, and questions 2, 5, 7, 8, and 10 in all but one. Questions 2, 6, and 11 were not answered on 2 occasions, and questions 9 and 12 on 3 occasions. OKS questions 7, 9, and 12 were answered in all cases, and questions 1, 2, 3, 5, 6, 10, and 11 in all cases but one. Question 8 was not answered on 2 occasions, and question 4 in 6 cases. The EQ-5D-5 L was completed by all the patients. We obtained the complete OHS and OKS scores and the EQ-5D-5 L utility index for 347 patients with hip OA and 385 patients with hip OA. A follow-up was performed after 6 months of 313 patients with hip OA and 331 with knee OA, of whom 65 (20.8%) and 42 (12.7%) had undergone hip or knee replacement surgery, respectively. We obtained the complete OHS and OKS scores and the EQ-5D-5 l utility index for 301 and 316 patients with hip and knee OA, respectively, and their responses were used to validate the models (validation sample). Table 1 shows the patients’ characteristics.

**Table 1:** Characteristics of patients at inclusion and after 6 months

	Inclusion				6 Months			
	Total	Hip	Knee	***p***	Total	Hip	Knee	***p***
Total	758	361	397		644	313	331	
**Gender**								
Male N (%)	469 (61.9)	192 (53.2)	277 (69.8)	< 0.001	382 (59.3)	159 (50.8)	223 (67.4)	< 0.001
Female N (%)	289 (38.1)	169 (46.8)	120 (30.2)		262 (40.7)	154 (49.2)	108 (32.6)	
**Bilateral OA** N (%)	270 (35.6)	102 (28.3)	168 (42.3)	< 0.001	230 (35.7)	90 (28.8)	140 (42.3)	< 0.001
**Total Replacement** N (%)	135 (17.8)	63 (17.5)	72 (18.1)	0.806	229 (35.6)	121 (38.6)	108 (32.6)	0.110
**Age** Mean (SD)	69.7 (10.5)	67.9 (11.7)	71.4 (9.1)	< 0.001	70.2 (10.5)	68.3 (11.7)	71.9 (9.1)	< 0.001
**Charlson** Mean (SD)	0.808 (0.044)	0.836 (0.069)	0.782 (0.057)	0.271	0.785 (0.047)	0.792 (0.070)	0.778 (0.064)	0.440
**BMI** Mean (SD)	28.948 (0.175)	28.162 (0.243)	29.665 (0.247)	< 0.001	28.942 (0.188)	28.248 (0.259)	29.599 (0.268)	< 0.001
**EQ-5D-5 L**								
VAS	56.0 (21.9)	54.5 (22.3)	57.4 (21.6)	0.072	60.3 (22.5)	59.8 (22.9)	60.7 (22.1)	0.598
Utilities	0.53 (0.29)	0.52 (0.30)	0.54 (0.27)	0.252	0.60 (0.29)	0.60 (0.30)	0.60 (0.28)	0.797

**Fig. 1:**
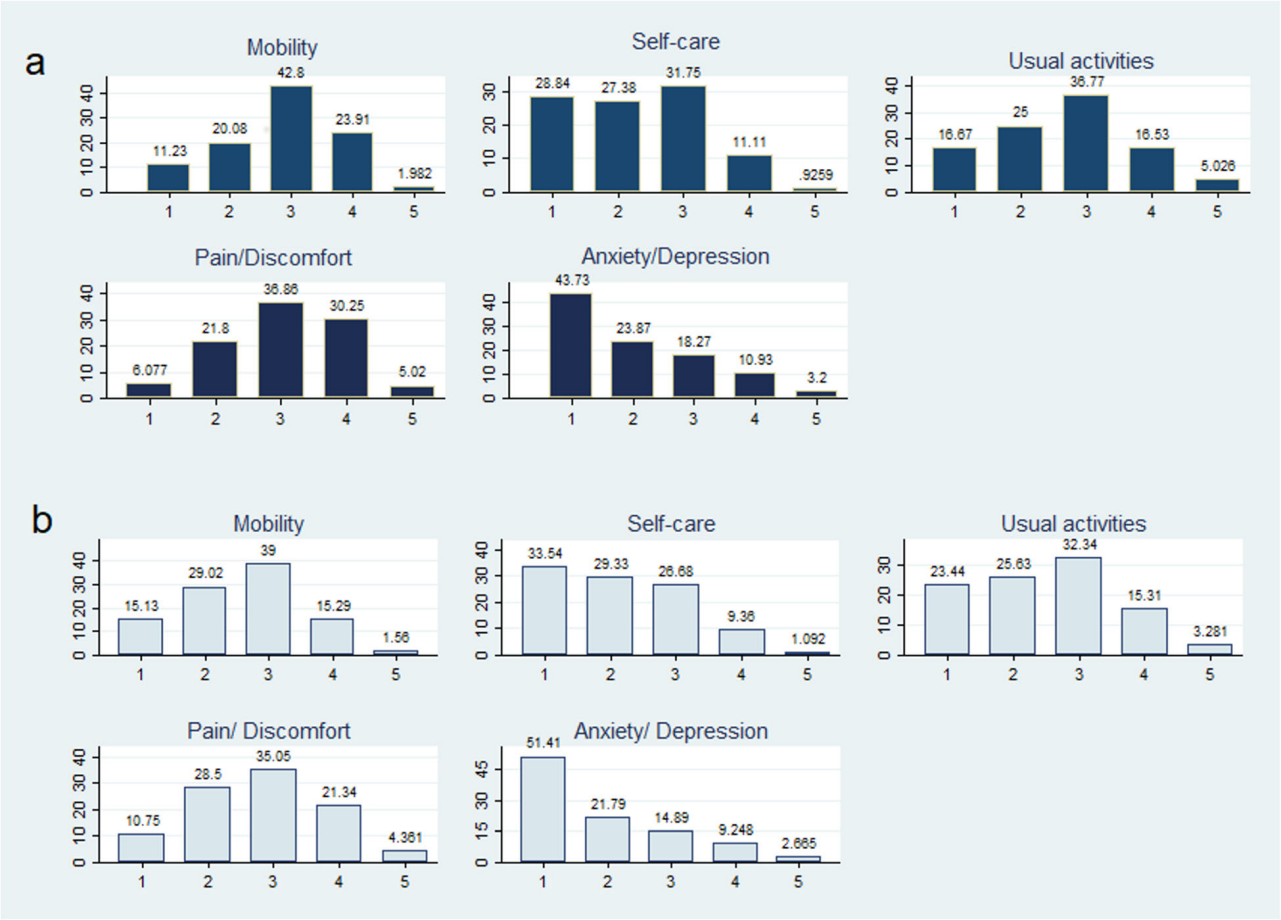
Response distribution for the 5 dimensions of EQ-5D-5 L, at inclusion (**1a**) and 6-month follow-up (**1b**)

Figure 1 shows the distribution of responses to the EQ-5D-5 L questionnaire at inclusion and 6 months later. The surveyed patients expressed 274 of the 3125 possible health conditions, with a utility range between − 0.416 and 1. The observed ceiling and floor effects for utilities were 2.5 and 0.3%, respectively.

The dimensions where patients reported the largest percentage of problems at the basal point were mobility (88.7%), performance of daily life activities (83.3%), and pain/discomfort (93.9%). However, 43.7% of the subjects reported not having problems when asked about the anxiety/depression dimension. All the dimensions showed improvement at the 6-month follow-up visit.

**Fig. 2:**
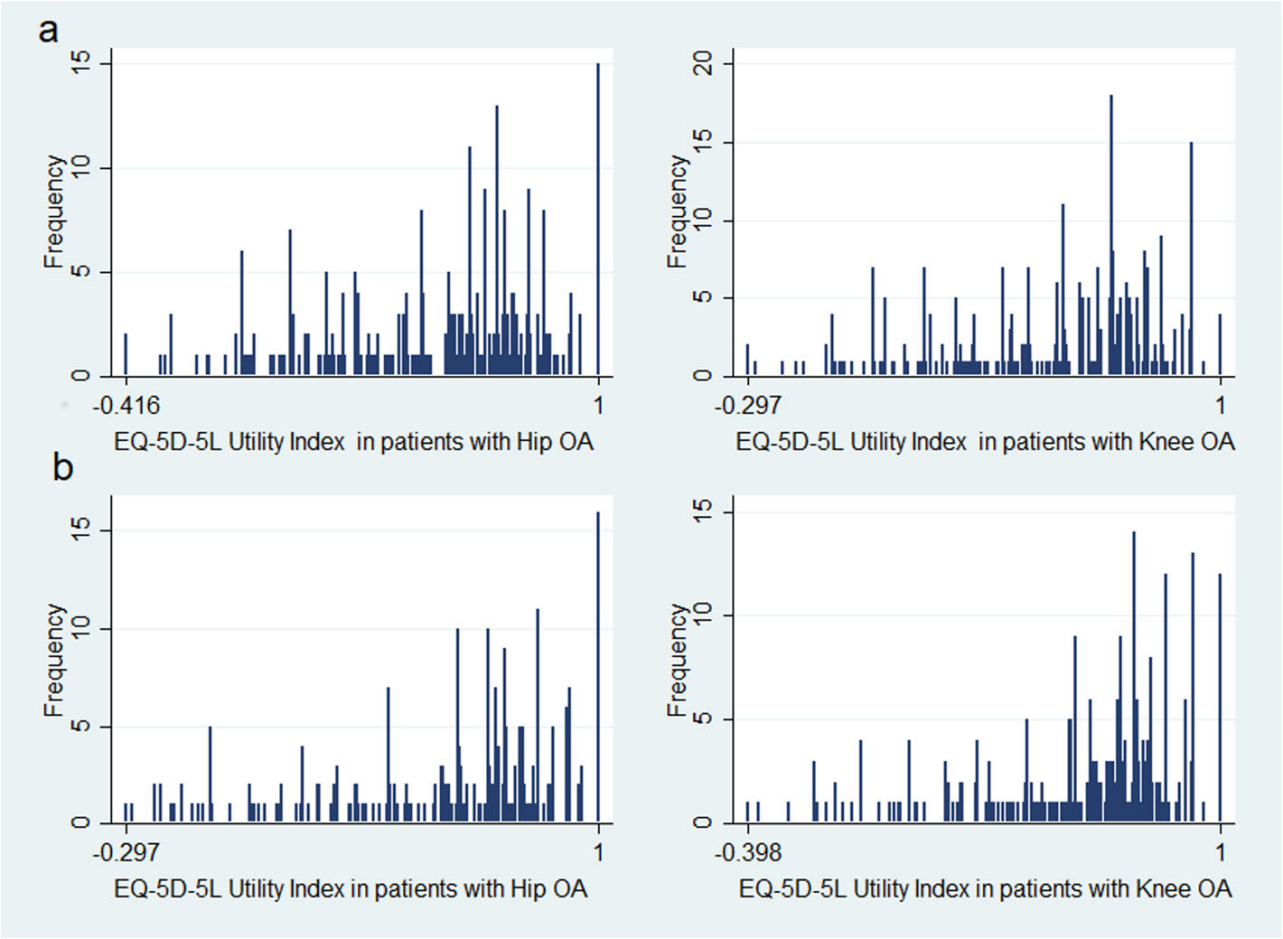
Utility indexes distribution for hip and knee OA, at inclusion (**2a**) and 6-month follow-up (**2b**)

Figure 2 shows the distribution of the obtained utility, which was the dependent variable selected for all the models. A considerable asymmetry to the right (positive) can be observed.

At the inclusion visit, a strong positive correlation was found between total scores on the OHS or OKS questionnaire and utility indexes. The OLS models used to test these relationships can be described as follows:

$$ {U}_i=-0.0211756+0.0237627\times OHSscore;{R}^2=0.697 $$

$$ {U}_i=0.0904995+0.0205973\times OKSscore;{R}^2=0.571 $$

In both cases, the residuals were approximately normally distributed.

### Evaluation of models

Responses from the OHS/OKS questionnaires could be treated as ordinal or continuous variables, under the assumption that they indicate levels of clinical severity [34]. As a result, we tested the best adjustment models for ordinal/continuous responses and ultimately selected the last option under the Bayes Information Criterion (BIC) performance for each model. The BIC values (full models with ordinal/continuous variables) were − 105.6/− 212.7, − 125.5/− 274.1, − 40.19/− 165.6 and − 560.8/− 657.5 for the OLS, Tobit model, GLM and beta regression in patients with hip OA, respectively. In patients with knee OA, the BIC values (full models with ordinal/continuous variables) were − 47.6/− 198.4, − 75.4/− 225.2, − 26.4/− 177.7 and − 287.4/− 383.7 for the OLS, Tobit model, GLM and beta regression, respectively.

### OHS mapping to EQ-5D-5 L

Patients with hip OA expressed 172 of the 3125 possible health conditions described by the EQ-5D-5 L, with a utility range of − 0.416 to 1 (Fig. 2 upper). The observed ceiling effect was 4.20%, and the floor effect was 0.56%.

Table 2 shows the statistically significant items of the OHS for predicting the expressed utilities for each model built. Age and sex were included as predictive variables in the preliminary tests but did not improve model fit in any case. Items not shown were excluded from the final models since they did not improve the models’ performance and had no effect on the coefficients of the included ones. Finally, 352 patients answered all the questions included in the models, and they made up the estimation sample.

**Table 2:** Coefficients of the predictive equations of utilities (EQ-5D) based on the Oxford Hip Score (OHS). Estimation sample

	OLS	Tobit	GLM	Beta regression
**Hip OA**^¶^				
OHS1: Usual level of pain		0.0400 ^Ŧ^ (0.0143)	−0.156^§^ (0.0374)	0.2346^Ŧ^ (0.0772)
OHS2: Difficulty with washing and drying	0.0578^§^ (0.0110)	0.0564^§^ (0.0107)	−0.0885^§^ (0.0219)	0.2080^§^ (0.0444)
OHS3: Difficulty with cars/public transport	0.0466^§^ (0.0129)	0.0561^§^ (0.0139)	−0.123^§^ (0.0343)	0.4317^§^ (0.0730)
OHS5: Grocery shopping alone			−0.0504* (0.0215)	0.0985* (0.0398)
OHS6: Walking duration before pain	0.0269^§^ (0.00768)	0.0262^§^ (0.0076)		0.0895* (0.0404)
OHS8: Pain from standing up from chair	0.0517^§^ (0.0118)	0.0402 ^Ŧ^ (0.0128)	−0.0633* (0.0289)	
OHS11: Pain that interferes with work	0.0941^§^ (0.0108)	0.0814^§^ (0.0115)	−0.165^§^ (0.0324)	0.2070^§^ (0.0521)
OHS12: Pain at night				0.0615* (0.0312)
Constant	−0.0292 (0.0292)	−0.0365 (0.0301)	0.232^§^ (0.0392)	−1.6057^§^ (0.2008)
Observations	352	351	352	350
Adjusted R^2^	0.697	0.715		
Sigma		0.168 (0.007)		
AIC	−256	− 213.4	−323	− 652.5
BIC	−232.8	− 182.9	− 295.9	− 617.8

^¶^ Questions 4,7,9, and 10 of the OHS did not fit any model

*OLS* Ordinary least squares

*GLM* Generalized linear model. Link function: log. Distributional family: Gaussian

Standard errors in parentheses

^*^p < 0.05

^Ŧ^*p* < 0.01

^§^*p* < 0.001

The interference of pain with usual work, having any trouble getting in and out of a car or using public transport, the usual level of pain, or difficulty washing or drying oneself were the items most strongly related to the utilities in the GLM and beta regression models. Interference of pain with work was the main predictive variable of utilities in the OLS and Tobit models. Questions referring to putting on a pair of socks, stockings or tights; walking up stairs; limping; or feeling acute pain due to hip osteoarthritis were not related to express utilities in any model.

### OKS mapping to EQ-5D-5 L

Patients with knee OA expressed 180 of the 3125 possible health conditions described in the EQ-5D-5 L, with reported utilities in the range of − 0.297 to 1 (Fig. 2 upper). The maximum possible score (score of 1) was expressed by 1.02% of patients, and 0.51% reported the minimum score.

Table 3 shows the built models, with the statistically significant OKS items to predict utilities for patients with knee OA. Age and sex were also included as predictive variables in the preliminary tests but did not improve model performance.in any case. Items not shown were excluded from the final models since they did not improve the model fit and had no effect on the coefficients of the included ones. Finally, 390 patients answered all the questions included in the models, and they made up the estimation sample.

**Table 3:** Coefficients of the predictive equations of utilities (EQ-5D) based on the Oxford Knee Score (OKS) items. Estimation sample

	OLS	Tobit	GLM	Beta regression
**Knee OA**^¶^				
OKS1: Usual level of pain	0.0471^§^ (0.0122)	0.0434^§^ (0.0129)	−0.155^§^ (0.0384)	0.2227^Ŧ^ (0.0699)
OKS2: Difficulty with washing and drying	0.0532^§^ (0.0122)	0.0525^§^ (0.0121)	−0.106^§^ (0.0271)	0.2296^§^ (0.0521)
OKS3: Difficulty with cars/public transport				0.1592* (0.0666)
OKS4: Walking duration before pain				0.0794* (0.0353)
OKS9: Pain that interferes with work	0.0574^§^ (0.0119)	0.0515^§^ (0.0121)	−0.120^§^ (0.0317)	0.2688^§^ (0.0477)
OKS10: Knee instability sensation	0.0289^Ŧ^ (0.0102)	0.0263^Ŧ^ (0.0100)	−0.0491* (0.0207)	0.1431^§^ (0.0340)
OKS11: Grocery shopping alone	0.0432^§^ (0.0104)	0.0385^§^ (0.0104)	−0.0710^Ŧ^ (0.0235)	
OKS12: Difficulty walking down stairs		0.0276* (0.0118)	−0.0881^Ŧ^ (0.0280)	
Constant	0.122^§^ (0.0270)	0.1034^§^ (0.0273)	0.124* (0.0536)	−1.4199^§^ (0.2001)
Observations	390	390	390	386
Adjusted R^2^	0.595	0.606		
Sigma		0.171 (0.007)		
AIC	− 257.1	−211.3	−289.1	− 457.2
BIC	−233.3	−243.1	−261.3	− 425.6

¶ Questions 5, 6, 7 and 8 of the OKS did not fit any model

*OLS* Ordinary least squares

*GLM* Generalized linear model. Link function: log. Distributional family: Gaussian

Standard errors in parentheses

* *p* < 0.05

Ŧ *p* < 0.01

§ *p* < 0.001

The interference of pain with typical work, having any trouble washing or drying oneself, and the usual level of pain, were the items most strongly related to the utilities in the GLM and beta regression models. Trouble walking down stairs was also relevant in the GLM model. Questions referring to feeling pain after standing up from a chair, limping, being able to kneel and get up again afterwards and feeling pain at night were not related to expressed utilities in any model.

Table 4 presents the fit for each of the built models to predict expressed utilities in the estimation sample. GLM models and beta regressions had the smallest errors, and the highest level of agreement for estimates.

**Table 4:** Error measurements for predicting utility values based on OHS and OKS questionnaires using the different models. Estimation sample

		MAE	MSE	SE^a^	ICC
Model	Dependent Variable				
**Hip osteoarthritis, OHS,*n*** **= 352**					
OLS	Utility	0.1263 (0.1157–0.1368)	0.0259 (0.0217–0.0301)	0.0200 (0.0194–0.0207)	0.825 (0.789–0.856)
Tobit	Utility	0.1228 (0.1125–0.1331)	0.0247 (0.0206–0.0288)	0.0223 (0.0216–0.0231)	0.832 (0.797–0.862)
GLM	Utility	0.1156 (0.1058–0.1256)	0.0222 (0.0184–0.0260)	0.0216 (0.0204–0.0227)	0.856 (0.826–0.882)
Beta reg	Utility	0.1199 (0.1093–0.1304)	0.0244 (0.0200–0.0288)	0.0067 (0.0066–0.0069)	0.861 (0.832–0.886)
**Knee Osteoarthritis, OKS,*n*** **= 390**					
OLS	Utility	0.1340 (0.1232–0.1448)	0.0297 (0.0249–0.0343)	0.0210 (0.0204–0.0216)	0.750 (0.703–0.790)
Tobit	Utility	0.1313 (0.1206–0.1421)	0.0296 (0.0242–0.0334)	0.0221 (0.0216–0.0228)	0.751 (0.705–0.792)
GLM	Utility	0.1248 (0.1140–0.1356)	0.0272 (0.0224–0.0319)	0.0228 (0.0218–0.0239)	0.774 (0.731–0.811)
Beta reg	Utility	0.1287 (0.1176–0.1397)	0.0287 (0.0240–0.0335)	0.0075 (0.0074–0.0076)	0.798 (0.769–0.823)

*MAE* Mean absolute error, *MSE* Mean squared error

*SE*^a^: Standard error reported are the mean values for the original predictions (disutility for GLM and transformed utility (0–1) for beta reg)

CI 95% in parentheses

*ICC* Intraclass correlation coefficient. Observed-predicted values (absolute agreement)

### Validation of predictive equations

The sample of subjects with hip OA expressed utilities slightly higher at 6 months than at the inclusion visit (0.075 points; CI 95%: 0.029–0.121), reporting 153 different health conditions, with a utility range between − 0.297 and 1 (Fig. 2 lower). The best possible health condition was expressed by 5.14% of the patients, and no floor effect was observed.

In the assessment of patients with knee OA at 6 months, the utility index also showed improvement (0.057 points; CI 95%: 0.016–0.097), ranging from − 0.398 to 1 and expressing 146 different health conditions (Fig. 2 lower). The observed ceiling effect was 3.70%, and no aggregation was found among the lower scores.

The mean (SD) for the observed utility values was 0.5949 (0.3012) in patients with hip OA, and the means for predicted values were 0.5790 (0.2813), 0.5842 (0.2748), 0.5821 (0.2613) and 0.5916 (0.3051) for the OLS, Tobit model, GLM and beta regression, respectively.

In patients with knee OA, the mean (SD) for the observed utility values was 0.6008 (0.27923), and the predicted mean utility values were 0.6065 (0.2339), 0.5994 (0.2243), 0.5969 (0.2277) and 0.5986 (0.2398) for the OLS, Tobit model, GLM and beta regression, respectively.

Table 5 shows the fit for each of the built models to predict expressed utilities in the validation sample. In terms of MAEs, GLM and Breg were the best predictive models for both hip and knee OA. In terms of measurement variability, all the predictions were acceptably accurate; the Breg SE coefficients were significantly lower than the outcomes of the other models but direct comparisons cannot be made as the utility variable was transformed for Breg. The ICCs were around the milestones of 0.8 in all cases but were slightly better for Breg and GLM.

**Table 5:** Error measurements for predicting utility values based on OHS and OKS questionnaires using the different models. Validation sample

		MAE	MSE	SE^a^	ICC
Model	Dependent Variable				
**Hip osteoarthritis, OHS,*n*** **= 301**					
OLS	Utility	0.1343 (0.1215–0.1471)	0.0307 (0.0248–0.0365)	0.0206 (0.0200–0.0213)	0.817 (0.775–0.851)
Tobit	Utility	0.1265 (0.1143–0.1387)	0.0275 (0.0220–0.0330)	0.0246 (0.0238–0.0254)	0.833 (0.794–0.864)
GLM	Utility	0.1103 (0.0993–0.1214)	0.0216 (0.0167–0.0264)	0.0212 (0.0197–0.0227)	0.855 (0.821–0.882)
Beta reg	Utility	0.1229 (0.1102–0.1335)	0.0274 (0.0211–0.0338)	0.0067 (0.0065–0.0070)	0.850 (0.815–0.878)
**Knee Osteoarthritis, OKS,*n*** **= 316**					
OLS	Utility	0.1278 (0.1159–0.1398)	0.0279 (0.0228–0.0331)	0.0205 (0.0199–0.0211)	0.788 (0.743–0.826)
Tobit	Utility	0.1236 (0.1117–0.1355)	0.0268 (0.0216–0.0320)	0.0219 (0.0213–0.0225)	0.791 (0.746–0.829)
GLM	Utility	0.1127 (0.1014–0.1239)	0.0230 (0.0181–0.0277)	0.0204 (0.0192–0.0215)	0.824 (0.785–0.856)
Beta reg	Utility	0.1141 (0.1031–0.1251)	0.0229 (0.0186–0.0272)	0.0063 (0.0062–0.0064)	0.832 (0.795–0.863)

*MAE* Mean absolute error, *MSE* Mean squared error

*SE*^a^ Standard error reported are the mean values for the original predictions (disutility for GLM and transformed utility (0–1) for beta reg)

CI 95% in parentheses

*ICC* Intraclass correlation coefficient. Observed-predicted values (absolute agreement)

Table 6 shows the MAE values obtained for the bottom half (expressed utility < median) and the top half (expressed utility ≥ median) of the scale. The median utility values were 0.6973 and 0.6852 for patients with hip and knee OA, respectively. The performance of the models was similar, but consistent differences were found for the bottom and top halves of the scale for each model.

**Table 6:** Error measurements for predicting utility values based on OHS and OKS, according to the distribution of expressed utilities. Validation sample

		**Hip osteoarthritis, OHS,*n*** **= 301**	
		MAE	
		Utility score ≥ median^a^	Utility score < median^a^
Model	Dependent Variable		
OLS	Utility	0.1137 (0.0981–0.1293)	0.1576 (0.1374–0.1777)
Tobit	Utility	0.1031 (0.0885–0.1177)	0.1518 (0.1325–0.1711)
GLM	Utility	0.0857 (0.0742–0.0973)	0.1410 (0.1213–0.1711)
Beta reg	Utility	0.0941 (0.0801–0.1080)	0.1536 (0.1330–0.1741)
		**Knee osteoarthritis, OKS,*n*** **= 313**	
		MAE	
		Utility score ≥ median^b^	Utility score < median^b^
Model	Dependent Variable		
OLS	Utility	0.1106 (0.0969–0.1243)	0.1470 (0.1279–0.1661)
Tobit	Utility	0.1031 (0.0899–0.1164)	0.1451 (0.1259–0.1642)
GLM	Utility	0.0869 (0.0758–0.0980)	0.1389 (0.1204–0.1575)
Beta reg	Utility	0.0899 (0.0782–0.1016)	0.1389 (0.1208–0.1570)

^a^Median utility value (Hip osteoarthritis): 0.6973

^b^Median utility value (Knee osteoarthritis): 0.6852

*OLS* Ordinary least squares

*GLM* Generalized linear model. Link function: log. Distributional family: Gaussian

*Beta reg* Beta regression

*MAE* Mean absolute error

CI 95% in parentheses

No differences were found in model performance for predicting utilities in subjects which had undergone an intervention for joint replacement.

**Fig. 3:**
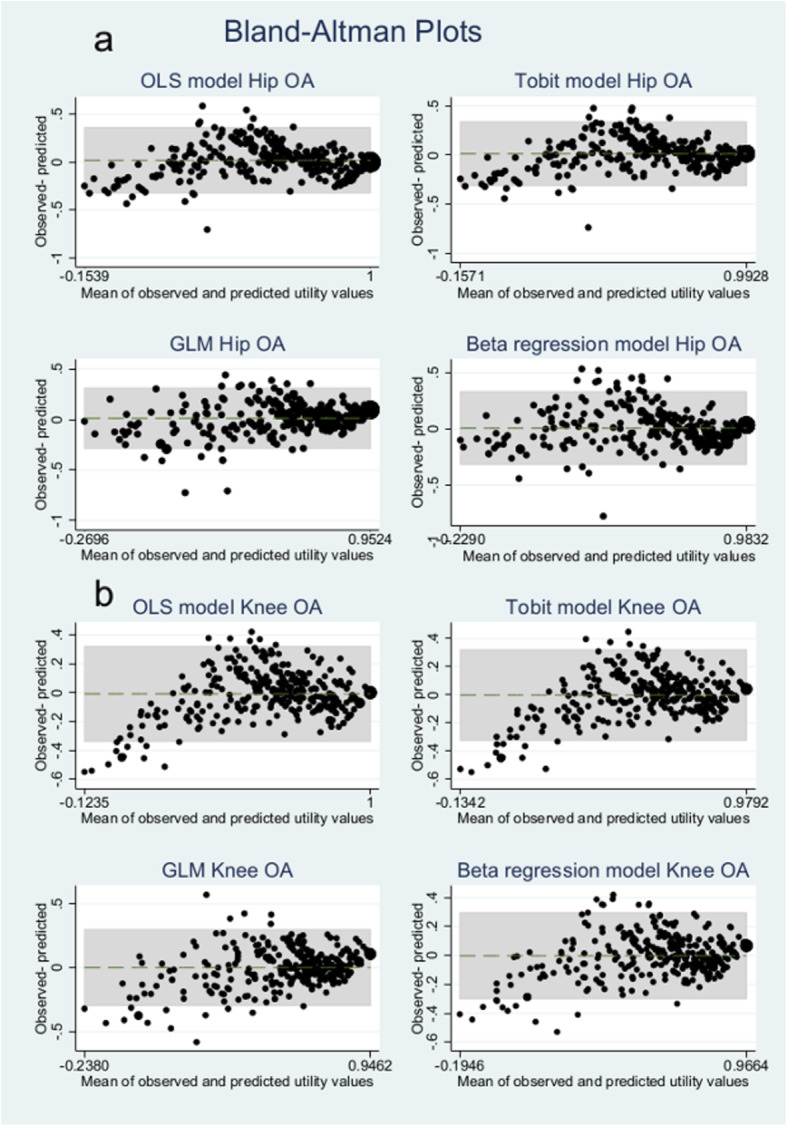
Bland-Altman plots for predicted and observed utility values based on OHS (**3a**) or OKS (**3b**) scores

Figure 3 shows the generated Bland-Altman plots comparing the observed and predicted values resulting from each method and graphically shows adequate agreement between them, although dispersion increased in the lower part of the utilities’ distribution.

As GLM and beta regression appeared to generate the best predictive models, the prediction of utilities based on OHS and OKS scores could be performed as follows:

- GLM:

Step 1. Estimation of the ln of disutility (A).

OHS

$$ A=0.232-0.156\times OHS1-0.885\times OHS2-0.123\times OHS3-0.0504\times OHS5-0.0633\times OHS8-0.165\times OHS11 $$

OKS

$$ A=0.124-0.155\times OKS1-0.106\times OKS2-0.120\times OKS9-0.0491\times OKS10-0.0710\times OKS11-0.0881\times OKS12 $$

Step 2. Estimation of the EQ-5D-5 L utility index to the possible range of the real index in the sample.

$$ \mathrm{Predicted}\kern0.5em \mathrm{EQ}-5\mathrm{D}-5\mathrm{L}\kern0.5em \mathrm{utilityindex}=1-{e}^A $$

b) Beta regression:

Step 1. Estimation of the logit of the transformed EQ-5D-5 L utility index to the (0, 1) open unit interval (A).

OHS

$$ A=-1.6057+0.2346\times OHS1+0.2080\times OHS2+0.4317\times OHS3+0.0985\times OHS5+0.0895\times OHS6+0.2070\times OHS11+0.0615\times OHS12 $$

OKS

$$ A=-1.4199+0.2227\times OKS1+0.2296\times OKS2+0.1592\times OKS3+0.794\times OKS4+0.2688\times OKS9+0.1431\times OKS10 $$

Step 2. Estimation of the transformed EQ-5D-5 L utility index to the (0, 1) open interval.

$$ B={e}^A/\left(1+{e}^A\right) $$

Step 3. Estimation of the EQ-5D-5 L utility index to the possible range of the real index in the sample.

$$ \mathrm{Predicted}\kern0.5em \mathrm{EQ}\hbox{-} 5\mathrm{D}\hbox{-} 5\mathrm{L}\kern0.5em \mathrm{utility}\kern0.5em \mathrm{index}=\left(1-\min .\kern0.5em value\right)\times B+\min .\kern0.5em value $$

## Discussion

The OHS and OKS questionnaires outcomes are useful for predicting utility scores expressed by patients with hip and knee OA, respectively. The proposed equations allow for making valid and precise predictions using the independent variables from Tables 2 and 3. Notably, the results of these equations stem from responses that reflect information on patients with a broad range of clinical stages.

In terms of apparent validity, it is noteworthy that the models are congruent, since the higher the scores of the OKS/OHS items are, the higher the utility level is, and as described above, total OHS/OKS scores are strong predictors of utilities [27, 28].

For patients with hip OA, the coefficients with the highest predictive capacity are related to OHS questions about pain (usual level of pain), self-care (trouble washing or drying oneself), mobility (difficulty with cars/public transport, or walking duration before pain), and functionality (interference of pain with typical work).

Questions rejected in all the OHS models (difficulty putting on a pair of socks, stockings or tights, walking up stairs, limping or feeling acute pain due to hip osteoarthritis) only partially overlap with questions without significance in other studies [28].

This pattern was also found for patients with knee OA. Questions referring to pain (usual level of pain), self-care (difficulty washing or drying oneself), mobility (difficulty with cars/public transport, or walking duration before pain – only in Breg), and functionality (interference of pain with typical work) were the items most strongly related to the utilities in Breg and GLM.

These findings are not surprising as pain/discomfort, self-care, mobility and daily life performance (which could be described as functionality) are four of the five dimensions of the EQ-5D-5 L. It has been debated whether scores from the OKS or OHS questionnaires yield adequate utility predictions similar to those proposed by the EQ-5D-5 L for health conditions [40]. Two different factor structures have been proposed for the OHS/OKS, the first assessing a single dimension [25, 26, 34, 41] and the second including two factors, pain and functionality [42, 43]. The second proposal for both the OHS and OKS comprises questions on functionality whose apparent validity can relate this domain to self-care and mobility. Therefore, a substantial overlap can exist in the construct assessed via both types of questionnaires, although the EQ-5D-5 L dimension on anxiety/depression is not specifically addressed by the OHS/OKS questionnaires, which could limit their ability to predict EQ-5D-5 L utility values [40]. Nevertheless, the OKS questionnaire has been found to predict anxiety/depression responses reasonably accurately, probably because pain and poor knee function explain much of the anxiety/depression observed in this population [27].

One item (pain at night) was discarded during the iterative process for generating the best equation, whereas the sign of the association was the opposite of that expected from the OLS and Tobit models. The magnitude of the association with utilities was negligible (~ − 0.015), and removing this item from the models improved the adjustment indexes (AIC and BIC). This problem was not present using the GLM or Breg. Frequent difficulty with apparent validity has been documented in studies with similar objectives to ours [27] and when mapping from general questionnaires to the EQ-5D [44].

Once face validity appears appropriate, the selection of the best model should be discussed.

One aspect to be considered is the possibility of treating predictive variables as continuous or ordinal. Responses to OKS/OHS questionnaires are usually made using Likert scales [42, 43, 45, 46]. Instruments that use Likert-type responses provide a categorical description of an underlying continuous variable. The use of parametric statistics with Likert data with small sample sizes, unequal variances, and non-normal distributions has been supported with experimental designs [47]. Some studies have noted a better fit treating predictive variables as ordinal [48], but our study chose a continuous distribution given the fitting results under the BIC criteria. It should be noted that OHS and OKS indexes are obtained as the sum of their values, and the validation process of these questionnaires in their original [23, 24] and Spanish-adapted [25, 26] versions has treated responses as continuous values, showing excellent psychometric properties in both cases.

When looking for the best model, we found that the statistical models explained 60–70% of the response variability of perceived utilities, a similar value to those found in other mapping analyses using the same tools in English populations [28]. Correlation between observed and predicted values of utilities was strong and around the cut-off point of 0.8; this is an excellent result highlighting that total agreement coefficients were tested, which suggests that the means and variances of distribution were similar [49]. MAE values were lower than those reported for similar mapping procedures for knee OA [27] and equal to or lower than those reported in hip OA [28]. MSE values were meaningfully lower than those reported in previous mapping exercises in patients with hip [28] or knee OA [27] when we look at GLM and at Breg. These two models performance was similar to the observed one for other mapping exercises from WOMAC on to EQ-5D utilities in Spanish patients [50], which suggests that OHS and OKS are useful instruments for predicting utilities in patients with lower limb osteoarthritis.

GLM and Breg error measures were also lower than those reported by other authors performing mapping exercises on the EQ-5D with other types of illness [51]. GLM and Breg turned out to be the most accurate methods for predicting EQ-5D utilities from OHS and OKS scores in patients with lower limb OA in Spain, and its performance was much better than, for example, OLS methods, proposed by other authors [48].

Accuracy of the predicted measures was greater for patients with better health status as seen in the Bland-Altman plots for all the models. These plots show an overprediction for very severe health states (utility index less than 0). So, the observed MAE values were greater for the bottom half of the utilities scale but lower than those found in other OHS mapping analyses, which reported values of predicted utility scores in the range of 0.20–0.23 below 0.5 on the utility scale and 0.10–0.13 above 0.5 on the utility scale, the latter being more similar to values found in this study [28]. Compared to OKS reported values, other studies analysing similar predictive models with larger sample sizes and a narrower spectrum of the disease [27] found lower MAE and MSE values, with better health status independent of the chosen statistical model. This fact has implications for assessing the validity of predictions, since the prediction error seems to increase for patients reporting worse health conditions.

This study shows the same limitations as other mapping studies. Whether utilities obtained using mapping functions fit real observed values has been debated. There are studies showing the validity of this outcome compared to utilities directly assigned to the same health conditions [52], which supports the use of these methods. It is well established that mapping results in information loss and increased uncertainty and that direct EQ-5D measurements are preferable to mapping exercises, but the latter is frequently the only feasible way to conduct cost-utility analyses in cases where direct evidence is unavailable [6].

The validation process was performed on a sample evaluated at a different time. The dataset for validation was entirely different from that used to assess the models, even if it stemmed from the same patients. Nevertheless, additional external validation processes may have been necessary. It could be assumed that the studied sample represents the spectrum of patients who use the National Health System to obtain care for their lower limb OA, patients included covered a wide range of disease severity, as shown by the wide distribution of the utility index and the health conditions represented, in addition to the fact that patients came from different regions and levels of health care. Consequently, the results should be useful for cost-utility studies in these patients in Spain, especially when their health status is not yet deeply affected.

## Conclusions

The scores from each item of the OHS and OKS questionnaires allowed for estimating EQ-5D-5 L utilities in patients with hip and knee OA, respectively, with adequate precision. The GLM and Breg models were the best approach to predict EQ-5D utilities in patients with lower limb OA. Prediction of utility values was more consistent for subjects in better health. Therefore, further research on prediction models for subjects in poor health is recommended.

## Supplementary information

**Additional file 1.** Spanish-adapted version of the Oxford Hip Score - Spanish (Spain).

**Additional file 2.** Spanish-adapted version of the Oxford Knee Score - Spanish (Spain).

**Additional file 3 Table 1.** Checklist of Items to Include When Reporting a Mapping Study.

## Data Availability

The datasets used or analysed during the current study are available from the corresponding author upon reasonable request.

## Abbreviations

AIC: Akaike Information Criteria
BIC: Bayes Information Criteria
Breg: Beta regression
CI 95%: 95% Confidence Interval
DALY: Disability-adjusted life years
HRQoL: Health-Related Quality of Life
GLM: Generalized Linear Model
ICC: Intraclass Correlation Coefficient
MAE: Mean absolute error
MSE: Mean squared error
OA: Osteoarthritis
OHS: Oxford Hip Score
OKS: Oxford Knee Score
OLS: Ordinary Least Squares
SE: Standard Error
WOMAC: Western Ontario and McMaster Universities Osteoarthritis Index
